# First results of the uveitis outcome study of the multinational interdisciplinary working group for uveitis in childhood (MIWGUC)

**DOI:** 10.1186/1546-0096-12-S1-P50

**Published:** 2014-09-17

**Authors:** Ivan Foeldvari, Gabriele Brumm, C Delibero, Kerstin Gerhold, Valeria Maria Gerloni, Kaisu Kotaniemi, Kirsten Minden, Eilsabeth Miserochchi, Uwe Pleyer, Gabriele Simonini, Susan Nielsen, I Sandfeldt, Martina Niewerth, Sandra Schenck, Arnd Heiligenhaus

**Affiliations:** 1 Pediatric Rheumatology, Hamburg, Germany; 2 Ophthalmology, Hamburg, Germany; 3 Ophthalmology, Florence, Italy; 4 DRFZ, Berlin, Germany; 5 Pediatric Rheumatology, Milan, Italy; 6 Ophthalmology, Helsinki, Finland; 7 Charitee, Berlin, Germany; 8 Ophthalmology, Milan, Italy; 9 Pediatric Rheumatology, Florence, Italy; 10 Pediatric Rheumatology, Copenhagen, Denmark; 11 Ophthalmology, Copenhagen, Denmark; 12 Ophthalmology, Münster, Germany

## Introduction

Juvenile idiopathic arthritis (JIA) associated uveitis is one of the most severe comorbidities of JIA and it occurs in about 10 to 20 % of JIA patients. There are currently no specific established outcome measures for this specific uveitis. The Standardization of Uveitis Nomenclature SUN* group made the first attempt to establish outcome measures for uveitis in adults (1). We adapted part of it and developed and proposed outcome measures over a consensus process specific for JIA associated uveitis (2). Here we present the first results of the prospective evaluation of this outcome measures.

## Objectives

To validate the proposed outcome measures for JIA associated uveitis

## Methods

Patients were enrolled, at a start of the treatment with a nonbiologic or biologic disease modifying agents due to the severity of the uveitis and were followed with the proposed parameters to asses changes of the uveitis. The parameters evaluated at each visit included: demographics, rheumatologic assessment (JIA type, activity of arthritis, JIA-related disability), ophthalmologic assessment (duration of uveitis, activity of uveitis,visual acuity, ocular complications, topical and systemic medications, surgical procedure, uveitis-related disability). Quality of life questionnaire for parents and children were also assessed throughout the study.

## Results

At present 33 patients completed the first 3 months of the follow-up. 61% of them were female. Mean age at inclusion into the study was 8 years. 97% of the patients were Caucasian. The JIA subset distribution was 68% oligoarticular, 16% extended oligoarticular, 12% RF negative polyarticular and 4% enthesitis-related. At enrolment mean disease duration of JIA was 53 months and the uveitis 33 months. On a VAS-score(0-100) the uveitis related disability was 20 and the JIA related disability 30. Insidious anterior uveitis was found at 95% of the patients at baseline , had, the left eye was involved more frequently ( 97.%) compared to 79% the right eye, in some patients both eyes were involved. Number of patients with more then 6 cells in the anterior chamber dropped from around 60% at baseline to 13% at months 3. A flare faint or more severe was observed in 75% of the eyes at baseline and in 30% at months 3. The anterior chamber flare grade according to the MIWGUC group responded quite well, at time point 0 over 80% had a flare and after 3 months only around 30%.

## Conclusion

These preliminary results of the standardized assessment of the JIA associated uveitis are promising. Further evaluation of these items will probably help to establish standardized measures to assess the activity of uveitis and the efficacy of a drug involved in the treatment.

## Disclosure of interest

None declared.

